# Supplementary material for: Analysis of PPARγ Signaling Activity in Psoriasis
Source: Int J Mol Sci. 2021 Aug 10;22(16):8603. doi: 10.3390/ijms22168603 (PMC8395241; doi:10.3390/ijms22168603)
Supplement: Supplementary file 1 [file ijms-22-08603-s001.zip › Supplemental materials_Analysis of PPARg signaling activity in psoriasis/Pathway models/Models images and html files/Interleukin-17 and interleukin-22 signaling in psoriasis/10342.html]

CCL2


# Protein CCL2

|  |  |
| --- | --- |
| URN | urn:agi-llid:6347 |
| Total Entities | 0 |
| Connectivity | 11154 |
| Name | CCL2 |
| Description | chemokine (C-C motif) ligand 2 |
| Notes | This gene is one of several cytokine genes clustered on the q-arm of chromosome 17. Chemokines are a superfamily of secreted proteins involved in immunoregulatory and inflammatory processes. The superfamily is divided into four subfamilies based on the arrangement of N-terminal cysteine residues of the mature peptide. This chemokine is a member of the CC subfamily which is characterized by two adjacent cysteine residues. This cytokine displays chemotactic activity for monocytes and basophils but not for neutrophils or eosinophils. It has been implicated in the pathogenesis of diseases characterized by monocytic infiltrates, like psoriasis, rheumatoid arthritis and atherosclerosis. It binds to chemokine receptors CCR2 and CCR4. [provided by RefSeq, Jul 2013] |
| Primary Cell Localization | Extracellular |
| Class | Ligand |
| ObjectType | Protein |

---

|  |  |
| --- | --- |
| Pathway | CC Chemokine Receptor Signaling |
|  | Plasmin Effects in Inflammation |
|  | Proplatelet Maturation |
|  | Toll-like Receptor Independent Sterile Inflammation |
|  | Lipids Enhance Apoptotic Cell Engulfment and Reduce Inflammation |
|  | Keep out Signal: LTF Inhibits Neutrophil Recruitment and Inflammation |
|  | Eosinophil Survival by Cytokine Signaling |
|  | Mast-Cell Activation without Degranulation through TNFSF8 Signaling |
|  | Mast-Cell Activation via IgE Signaling |
|  | Mast-Cell Activation without Degranulation |
|  | Mast-Cells De Novo Synthesized Mediators via IgE Independent Signaling |
|  | Vascular Endothelial Cell Activation by Cytokines |
|  | Vascular Endothelial Cell Activation by Blood Coagulation Factors |
|  | Adipokines Production by Adipocyte |
|  | Nociception Expression Targets Signaling |
|  | CCR1 Expression Targets |
|  | IL1B Expression Targets -> Nociception |
|  | Proteins Involved in Atherosclerosis |
|  | Scavenger Receptor OLR1 in Inflammation Related Endothelial Dysfunction |
|  | RAGE/AGER and S100 Proteins in Cardiovascular Injury |
|  | Low-Density Lipoproteins and Chemokines in Atherosclerosis |
|  | Chemokines Signaling in Atherosclerosis |
|  | Proteins Involved in Dilated Cardiomyopathy |
|  | Proteins Involved in Arterial Hypertension |
|  | Arterial Hypertension |
|  | Proteins Involved in Pulmonary Hypertension |
|  | Proteins Involved in Myocardial Ischemia |
|  | Proteins Involved in Myocarditis |
|  | IL17 Signaling in Psoriasis |
|  | Proteins Involved in Systemic Lupus Erythematosus |
|  | Toll-like Receptors in beta-Cell |
|  | TLR7 and TLR9 in Plasmacytoid Dendritic Cell |
|  | Proteins Involved in Diabetes Mellitus Type 1 |
|  | Proteins Involved in Diabetes Mellitus Type 2 |
|  | Endothelial Cell Dysfunction in Early Diabetic Nephropathy |
|  | Proteins Involved in Diabetic Nephropathy |
|  | Microangiopathy in Diabetic Neuropathy |
|  | Proteins Involved in Insulin Resistance |
|  | Adipose Tissue Inflammation Provokes Insulin Resistance |
|  | Hepatic Stellate Cells in non-Alcoholic Fatty Liver Disease |
|  | Proteins Involved in non-Alcoholic Fatty Liver Disease |
|  | beta-Cell Destruction through Cytokines in Diabetes Mellitus |
|  | Fatty Acids in Hypertension and Atherosclerosis Development |
|  | Adipokines Production by Adipocyte Impaired in Obesity |
|  | Proteins Involved in Obesity |
|  | Proteins Involved in Inflammatory Bowel Diseases |
|  | Mucosa-Associated Lymphoid Tissue (MALT) Lymphoma |
|  | Eosinophil Survival in Asthma |
|  | Mast-Cell Activation in Asthma |
|  | Neutrophil Recruitment in Sinovium in Gout |
|  | Proteins Involved in Polycystic Ovary Syndrome |
|  | Keratinocyte Activation in Psoriatic Arthritis |
|  | Proteins Involved in Rheumatoid Arthritis |
|  | Autocrine Cytokine/Chemokine Loops in Systemic Scleroderma |
|  | Th2-Cells Function in Systemic Scleroderma |
|  | Genes with Mutations Associated with Systemic Scleroderma |
|  | Proteins Involved in Systemic Scleroderma |
|  | Skin Fibrosis |
|  | Proteins with Altered Expression in Systemic Scleroderma |
|  | Proteins Involved in HIV Type 1 Infection |
|  | CCR5 Signaling in Macrophages |
|  | Estrogen Effects on Pregnancy Gingivitis |
|  | Majeed Syndrome |
|  | NF-kB Signaling Activation in Huntington Disease |
|  | Proteins Involved in Alzheimer's Disease |
|  | Proteins Involved in Epilepsy |
|  | Blood-Brain Barrier Disruption in Epileptiform Disorders |
|  | HMGB1 and IL1B in Neuroinflammation |
|  | ADK Expression Downregulation after Acute Seizures |
|  | Proteins Involved in Glioma |
|  | CCL2 Role in CGRP Expression and Release |
|  | Proteins with Altered Expression in Migraine |
|  | Proteins Involved in Neuroblastoma |
|  | Proteins Involved in Amyotrophic Lateral Sclerosis |
|  | Neuroinflammation in Amyotrophic Lateral Sclerosis |
|  | Proteins Involved in Multiple Sclerosis |
|  | Proteins Involved in Endometriosis |
|  | Coagulation Factors and PAR1/2 Receptors (F2R/F2RL1) in Endometriosis |
|  | Proteins Involved in Prostate Cancer |
|  | Proteins Involved in Age-Related Macular Degeneration |
|  | Proteins Involved in Glaucoma |
|  | Cochlear Inflammation (Noise-Induced) |
|  | Cytokines Trigger Otitis Media |
|  | Alveolar Macrophages Dysfunction in COPD |
|  | Proteins Involved in Chronic Obstructive Pulmonary Disease |
|  | Proteins Involved in Polycystic Kidney Disease |
|  | Proteins Involved in Glomerulonephritis |
|  | Plasmin Role in Glomerulonephritis |
|  | Endothelial Cell Dysfunction in Glomerulonephritis |
|  | S100B Expression Targets |
|  | Noradrenaline/Gs Expression Targets |
|  | Serotonin/Gs Expression Targets |
|  | Noreadrenaline/Gq Expression Targets |
|  | Serotonin/Gq Expression Targets |
|  | BMP7/BMPR2/ACVR2 Expression Targets |
|  | Elevated Receptors -> Expression Targets in Adipose Tissue |
|  | GPCR Family -> Expression Targets in Adipose Tissue |
|  | CTF1 Expression Targets |
|  | Elevated Receptors -> Expression Targets in Blood vessel |
|  | Elevated Receptors -> Expression Targets in Myocardium |
|  | Elevated Receptors -> Expression Targets in Blood Vessel |
|  | CXCL2 Expression Targets |
|  | CXCL5 Expression Targets |
|  | CCL3 Expression Targets |
|  | CCL16 Expression Targets |
|  | CSF2 -> NF-kB Expression Targets |
|  | CSF2 -> STAT Expression Targets |
|  | LIF Expression Targets |
|  | Cyclosporine Induced Nephrotoxicity |
|  | EGF -> CTNN Expression Targets |
|  | EGF -> AP-1/ATF Expression Targets |
|  | EGF -> CREB/CREBBP/ELK/SRF/MYC Expression Targets |
|  | EGF -> MEF/MYOD/NFATC Expression Targets |
|  | EGF -> STAT Expression Targets |
|  | Erythropoietin -> NF-kB Expression Targets |
|  | Erythropoietin -> AP-1/MYC/CREB Expression Targets |
|  | Erythropoietin -> STAT Expression Targets |
|  | Erythropoietin -> ELK/SRF Expression Targets |
|  | F2 -> AP-1/CREB/ELK/SRF/SP1 Expression Targets |
|  | F2 -> STAT1/NF-kB Expression Targets |
|  | FGF2 -> STAT Expression Targets |
|  | FGF2 -> AP-1/CREB/CREBBP/ELK/SRF/MYC Expression Targets |
|  | Glutamate/Gq Expression Targets |
|  | Elevated Receptors -> Expression Targets in Liver |
|  | Elevated Receptors -> Expression Targets in Mouth |
|  | GH1/GHR -> NF-kB/ELK/SRF/MYC Expression Targets |
|  | GH1/GHR -> STAT Expression Targets |
|  | GH1/PRLR Expression Targets |
|  | N2 Neutrophils in Tumor-Promoting Inflammation and Tumor Progression |
|  | HIF1A as Master Regulator of Angiogenesis in Cancer |
|  | Tumor Infiltrating Macrophages in Cancer Progression and Immune Escape |
|  | INHBA/ACVR2/BMPR Expression Targets |
|  | Insulin -> STAT Expression Targets |
|  | Insulin -> CEBPA/CTNNB/FOXA/FOXO Expression Targets |
|  | Insulin -> MEF/MYOD Expression Targets |
|  | Insulin -> ELK/SRF/HIF1A/MYC/SREBF Expression Targets |
|  | Fibronectin Expression Targets |
|  | CTGF -> AP-1/CREB/MYC Expression Targets |
|  | Fibrinogen Expression Targets |
|  | Elevated Receptors -> Expression Targets in Skin |
|  | IFNG/IFNR Expression Targets |
|  | IL13 Expression Targets |
|  | IL6 Expression Targets |
|  | IL2 Expression Targets |
|  | PTPRC -> BCL6 Expression Targets |
|  | Leptin -> STAT Expression Targets |
|  | Leptin -> ELK/SRF Expression Targets |
|  | Prostaglandin F Expression Targets |
|  | Thromboxane A2 Expression Targets |
|  | LPA Expression Targets |
|  | PAF/Gq -> AP-1/ATF1/CREB/ERK/SRF Expression Targets |
|  | PAF/Gq -> NF-kB Expression Targets |
|  | S1P Expression Targets |
|  | Proteins/Ions Transporters -> Expression Targets in Lymphoid System and Blood |
|  | Elevated Receptors -> Expression Targets in Lymphoid System and Blood |
|  | F2R/F2RL1 -> Expression Targets in Lymphoid System and Blood |
|  | GPCRs Family -> Expression Targets in Lymphoid System and Blood |
|  | TNF Receptors -> Expression Targets in Lymphoid System and Blood |
|  | Receptor Tyrosine Kinase Family -> Expression Targets in Lymphoid System and Blood |
|  | Elevated Receptors -> Expression Targets in Blood |
|  | CD40 -> Expression Targets in Thymus |
|  | P2RX7 -> Expression Targets in Nerve Tissue |
|  | Elevated Receptors -> Expression Targets in Nerve Tissue |
|  | NGF -> SMAD3/NF-kB Expression Targets |
|  | NGF -> AP-1/TP53/MYC Expression Targets |
|  | NGF -> FOXO/MYCN/ELK-SRF Expression Targets |
|  | NGF -> CREB/CEBPB/MEF2A Expression Targets |
|  | Adenosine Expression Targets |
|  | OSM/OSMR Expression Targets |
|  | PDGF -> AP-1/CREB/CREBBP/MYC Expression Targets |
|  | PDGF -> STAT Expression Targets |
|  | NTS Expression Targets |
|  | EDN1 Expression Targets |
|  | AGT -> CREB Expression Targets |
|  | IFNA1/Gq Expression Targets |
|  | AGT -> ELK/SRF Expression Targets |
|  | TAC1 Expression Targets |
|  | AGT -> STAT Expression Targets |
|  | PRL/PRLR Expression Targets |
|  | PRL/GHR -> STAT Expression Targets |
|  | PRL/GHR -> NF/kB/ELK/SRF/MYC Expression Targets |
|  | CSF1 -> STAT Expression Targets |
|  | MDK/PTN Expression Targets |
|  | Collagen -> NF-kB Expression Targets |
|  | PAF Expression Targets |
|  | CSF1 -> AP-1/CREB/CREBBP/MYC Expression Targets |
|  | PGF -> AP-1/CREB/CREBBP/MYC Expression Targets |
|  | TGFB2-TGFBR1 Expression Targets |
|  | TGFB1-TGFBR1 Expression Targets |
|  | TGFB2-TGFBR2 Expression Targets |
|  | TGFB1-TGFBR2 Expression Targets |
|  | TGFB1-TGFBR1/AP-1 Expression Targets |
|  | TGFB1-ACVRL1 Expression Targets |
|  | TNF -> NF-kB Expression Targets |
|  | TNFSF10 Expression Targets |
|  | LTA Expression Targets |
|  | TNFSF14 Expression Targets |
|  | TNF -> AP-1 Expression Targets |
|  | CD72 -> AP-1 Expression Targets |
|  | CD40LG -> NF-kB/ELK/SRF -> CREB/NFATC Expression Targets |
|  | TLR4 -> AP-1 Expression Targets |
|  | TLR7 Expression Targets |
|  | IL1B -> PGE2 Expression Targets |
|  | TLR1 -> 2/6 Expression Targets |
|  | IL1B Expression Targets |
|  | TLR4 -> NF-kB/IRF Expression Targets |
|  | IL1A Expression Targets |
|  | ROS in Triggering Vascular Inflammation |
|  | Elevated Receptors -> Expression Targets in Kidney |
|  | VEGFA -> ATF/CREB/ELK-SRF Expression Targets |
|  | VEGFA -> NFATC Expression Targets |
|  | VEGFA -> STAT Expression Targets |
|  | VEGFA -> AP-1/CREBBP/MYC Expression Targets |
|  | VEGFA -> CTNNB/CTNND Expression Targets |
|  | pathway 3 |
|  | e |
|  | pathway 4 |
|  | New Pathway (2) |
|  | cypa |
|  | pprarg neg, uknown targets, ps-positive |
|  | prarg neg,ukn expres targets, ps-positive |
|  | PPARG negative targets |
|  | pparg expr target\_regulators |
|  | Neighbors of disease exacerbation |
|  | Neighbors of tumor progression |
|  | Neighbors of cancer progression |
|  | Neighbors of inflammatory status |
|  | Neighbors of overall survival |
|  | Neighbors of disease severity |
|  | Neighbors of tumor size |
|  | Neighbors of lesion size |
|  | Neighbors of mortality |
|  | Neighbors of survival rate |
|  | Neighbors of infarct size |
|  | Neighbors of survival time |
|  | Neighbors of functional recovery |
|  | Neighbors of insulin sensitivity |
|  | Neighbors of bacterial load |
|  | Neighbors of cancer survival |
|  | Neighbors of graft survival |
|  | Neighbors of therapeutic efficacy |
|  | Neighbors of clinical stage |
|  | Neighbors of lethality |
|  | Neighbors of tumor regression |
|  | Neighbors of recurrence-free survival |
|  | Neighbors of tumor response |
|  | Neighbors of macrophage count |
|  | Neighbors of treatment outcome |
|  | Neighbors of bone mass |
|  | Neighbors of body weight |
|  | Neighbors of body weight gain |
|  | Neighbors of cardiovascular risk |
|  | Neighbors of microvessel density |
|  | Neighbors of tumor recurrence |
|  | Neighbors of tumor promotion |
|  | Neighbors of neutrophil count |
|  | Neighbors of glucose tolerance |
|  | Neighbors of morbidity |
|  | Neighbors of fibrosis progression |
|  | Neighbors of mortality rate |
|  | Neighbors of fat mass |
|  | Neighbors of exhaustion |
|  | Neighbors of bone mineral density |
|  | Neighbors of adverse outcome |
|  | Neighbors of lesion area |
|  | Neighbors of cell density |
|  | Neighbors of progression-free survival |
|  | Neighbors of apoptosis rate |
|  | Neighbors of early mortality |
|  | Neighbors of shear stress |
|  | Neighbors of viral load |
|  | Neighbors of blood pressure |
|  | Neighbors of leukocyte count |
|  | Neighbors of vascular reactivity |
|  | Neighbors of vascular leakage |
|  | Neighbors of body mass index |
|  | Neighbors of muscle mass |
|  | Neighbors of systolic blood pressure |
|  | Neighbors of tumor resistance |
|  | Neighbors of blood glucose |
|  | Neighbors of ejection fraction |
|  | Neighbors of fasting plasma glucose |
|  | Neighbors of osteoclast number |
|  | Neighbors of T-cell count |
|  | Neighbors of antimetastatic activity |
|  | Neighbors of glucose level |
|  | Neighbors of vascular tone |
|  | Neighbors of breast cancer risk |
|  | Neighbors of cell permeability |
|  | Neighbors of pregnancy outcome |
|  | Neighbors of neurological outcome |
|  | Neighbors of infarct area |
|  | Neighbors of recurrence rate |
|  | Neighbors of lymphocyte count |
|  | Neighbors of monocyte count |
|  | Neighbors of comorbidity |
|  | Neighbors of carotid intima-media thickness |
|  | Neighbors of fibroblast phenotype |
|  | Neighbors of fractional shortening |
|  | Neighbors of disease exacerbation |
|  | Neighbors of tumor progression |
|  | Neighbors of cancer progression |
|  | Neighbors of inflammatory status |
|  | Neighbors of overall survival |
|  | Neighbors of disease severity |
|  | Neighbors of tumor size |
|  | Neighbors of lesion size |
|  | Neighbors of mortality |
|  | Neighbors of survival rate |
|  | Neighbors of infarct size |
|  | Neighbors of survival time |
|  | Neighbors of functional recovery |
|  | Neighbors of insulin sensitivity |
|  | Neighbors of bacterial load |
|  | Neighbors of cancer survival |
|  | Neighbors of graft survival |
|  | Neighbors of therapeutic efficacy |
|  | Neighbors of clinical stage |
|  | Neighbors of lethality |
|  | Neighbors of tumor regression |
|  | Neighbors of recurrence-free survival |
|  | Neighbors of tumor response |
|  | Neighbors of macrophage count |
|  | Neighbors of treatment outcome |
|  | Neighbors of bone mass |
|  | Neighbors of body weight |
|  | Neighbors of body weight gain |
|  | Neighbors of cardiovascular risk |
|  | Neighbors of microvessel density |
|  | Neighbors of tumor recurrence |
|  | Neighbors of tumor promotion |
|  | Neighbors of neutrophil count |
|  | Neighbors of glucose tolerance |
|  | Neighbors of morbidity |
|  | Neighbors of fibrosis progression |
|  | Neighbors of mortality rate |
|  | Neighbors of fat mass |
|  | Neighbors of exhaustion |
|  | Neighbors of bone mineral density |
|  | Neighbors of adverse outcome |
|  | Neighbors of lesion area |
|  | Neighbors of cell density |
|  | Neighbors of progression-free survival |
|  | Neighbors of apoptosis rate |
|  | Neighbors of early mortality |
|  | Neighbors of shear stress |
|  | Neighbors of viral load |
|  | Neighbors of blood pressure |
|  | Neighbors of leukocyte count |
|  | Neighbors of vascular reactivity |
|  | Neighbors of vascular leakage |
|  | Neighbors of body mass index |
|  | Neighbors of muscle mass |
|  | Neighbors of systolic blood pressure |
|  | Neighbors of tumor resistance |
|  | Neighbors of blood glucose |
|  | Neighbors of ejection fraction |
|  | Neighbors of fasting plasma glucose |
|  | Neighbors of osteoclast number |
|  | Neighbors of T-cell count |
|  | Neighbors of antimetastatic activity |
|  | Neighbors of glucose level |
|  | Neighbors of vascular tone |
|  | Neighbors of breast cancer risk |
|  | Neighbors of cell permeability |
|  | Neighbors of pregnancy outcome |
|  | Neighbors of neurological outcome |
|  | Neighbors of infarct area |
|  | Neighbors of recurrence rate |
|  | Neighbors of lymphocyte count |
|  | Neighbors of monocyte count |
|  | Neighbors of comorbidity |
|  | Neighbors of carotid intima-media thickness |
|  | Neighbors of fibroblast phenotype |
|  | Neighbors of fractional shortening |
|  | Neighbors of cell development |
|  | Neighbors of immune response |
|  | Neighbors of cell population |
|  | Neighbors of T-cell development |
|  | Neighbors of inflammatory response |
|  | Neighbors of wound healing |
|  | Neighbors of angiogenesis |
|  | Neighbors of innate immune response |
|  | Neighbors of cell function |
|  | Neighbors of macrophage activation |
|  | Neighbors of cell formation |
|  | Neighbors of adaptive immune response |
|  | Neighbors of osteoclast development |
|  | Neighbors of epithelial to mesenchymal transition |
|  | Neighbors of immunity |
|  | Neighbors of cell proliferative response |
|  | Neighbors of chemotaxis |
|  | Neighbors of dendritic cell differentiation |
|  | Neighbors of cell survival |
|  | Neighbors of osteoclast differentiation |
|  | Neighbors of cell phenotype |
|  | Neighbors of T-cell proliferation |
|  | Neighbors of tissue remodeling |
|  | Neighbors of cell adhesion |
|  | Neighbors of T-cell activation |
|  | Neighbors of endothelial cell proliferation |
|  | Neighbors of fibrogenesis |
|  | Neighbors of tumor growth |
|  | Neighbors of dendritic cell development |
|  | Neighbors of tissue repair |
|  | Neighbors of cell count |
|  | Neighbors of cell infiltration |
|  | Neighbors of cell interaction |
|  | Neighbors of SMC proliferation |
|  | Neighbors of lumen formation |
|  | Neighbors of endothelial cell migration |
|  | Neighbors of adipogenesis |
|  | Neighbors of lipid storage |
|  | Neighbors of cell damage |
|  | Neighbors of pregnancy |
|  | Neighbors of monocyte differentiation |
|  | Neighbors of aging |
|  | Neighbors of cell motility |
|  | Neighbors of bone resorption |
|  | Neighbors of monocyte migration |
|  | Neighbors of fibroblast proliferation |
|  | Neighbors of adipocyte differentiation |
|  | Neighbors of macrophage migration |
|  | Neighbors of microglial activation |
|  | Neighbors of neutrophil recruitment |
|  | Neighbors of regeneration |
|  | Neighbors of cell migration |
|  | Neighbors of cancer growth |
|  | Neighbors of stem cell proliferation |
|  | Neighbors of smooth muscle cell migration |
|  | Neighbors of hepatic regeneration |
|  | Neighbors of transendothelial migration |
|  | Neighbors of phagocytosis |
|  | Neighbors of immune system activation |
|  | Neighbors of leukocyte migration |
|  | Neighbors of leukocyte recruitment |
|  | Neighbors of osteoclast formation |
|  | Neighbors of monocyte recruitment |
|  | Neighbors of cell invasion |
|  | Neighbors of heart function |
|  | Neighbors of hemopoiesis |
|  | Neighbors of bone remodeling |
|  | Neighbors of ossification |
|  | Neighbors of T-cell function |
|  | Neighbors of immune system function |
|  | Neighbors of lung development |
|  | Neighbors of autophagy |
|  | Neighbors of monocyte adhesion |
|  | Neighbors of transcription activation |
|  | Neighbors of senescence |
|  | Neighbors of T-cell response |
|  | Neighbors of tumor immunity |
|  | Neighbors of cell transdifferentiation |
|  | Neighbors of neuroprotection |
|  | Neighbors of stem cell differentiation |
|  | Neighbors of endothelial cell function |
|  | Neighbors of neuronal death |
|  | Neighbors of cell growth |
|  | Neighbors of ROS generation |
|  | Neighbors of epithelial cell proliferation |
|  | Neighbors of first trimester pregnancy |
|  | Neighbors of cancer cell growth |
|  | Neighbors of sensitization |
|  | Neighbors of hemato-encephalic barrier |
|  | Neighbors of macrophage function |
|  | Neighbors of cellular immune response |
|  | Neighbors of cellular senescence |
|  | Neighbors of colony formation |
|  | Neighbors of macrophage differentiation |
|  | Neighbors of cell differentiation |
|  | Neighbors of keratinocyte proliferation |
|  | Neighbors of stem cell migration |
|  | Neighbors of life span |
|  | Neighbors of psoriasis |
|  | Neighbors of inflammatory disease |
|  | Neighbors of rheumatoid arthritis |
|  | Neighbors of atherosclerosis |
|  | Neighbors of fibrosis |
|  | Neighbors of colitis |
|  | Neighbors of arthritis |
|  | Neighbors of inflammatory bowel disease |
|  | Neighbors of injury |
|  | Neighbors of atherogenesis |
|  | Neighbors of vascular remodeling |
|  | Neighbors of metastasis |
|  | Neighbors of carcinogenesis |
|  | Neighbors of multiple sclerosis |
|  | Neighbors of chronic inflammation |
|  | Neighbors of inflammation |
|  | Neighbors of tumor microenvironment |
|  | Neighbors of pathological angiogenesis |
|  | Neighbors of obesity |
|  | Neighbors of vascular disease |
|  | Neighbors of diabetes mellitus |
|  | Neighbors of asthma |
|  | Neighbors of pneumonia |
|  | Neighbors of hyperplasia |
|  | Neighbors of osteoarthritis |
|  | Neighbors of liver fibrosis |
|  | Neighbors of chronic obstructive pulmonary disease |
|  | Neighbors of myocardial infarction |
|  | Neighbors of leukocyte infiltration |
|  | Neighbors of reperfusion injury |
|  | Neighbors of airway inflammation |
|  | Neighbors of hepatitis |
|  | Neighbors of insulin resistance |
|  | Neighbors of infection |
|  | Neighbors of vascular injury |
|  | Neighbors of renal injury |
|  | Neighbors of preeclampsia |
|  | Neighbors of macrophage infiltration |
|  | Neighbors of liver injury |
|  | Neighbors of neuroinflammation |
|  | Neighbors of sepsis |
|  | Neighbors of death |
|  | Neighbors of type 1 diabetes |
|  | Neighbors of encephalomyelitis |
|  | Neighbors of autoimmune disease |
|  | Neighbors of ischemic stroke |
|  | Neighbors of dermatitis |
|  | Neighbors of neutrophil infiltration |
|  | Neighbors of heart disease |
|  | Neighbors of diabetic nephropathy |
|  | Neighbors of lung injury |
|  | Neighbors of immunopathology |
|  | Neighbors of systemic sclerosis |
|  | Neighbors of hepatocellular carcinoma |
|  | Neighbors of autoimmunity |
|  | Neighbors of acute lung injury |
|  | Neighbors of gastric cancer |
|  | Neighbors of atherosclerotic plaque |
|  | Neighbors of experimental autoimmune encephalomyelitis |
|  | Neighbors of colorectal cancer |
|  | Neighbors of cardiac remodeling |
|  | Neighbors of breast cancer |
|  | Neighbors of ischemia |
|  | Neighbors of systemic lupus erythematosus |
|  | Neighbors of melanoma |
|  | Neighbors of collagen-induced arthritis |
|  | Neighbors of cartilage degeneration |
|  | Neighbors of interstitial fibrosis |
|  | Neighbors of neoplasm invasion |
|  | Neighbors of kidney disease |
|  | Neighbors of heart failure |
|  | Neighbors of lung disease |
|  | Neighbors of pulmonary fibrosis |
|  | Neighbors of cardiovascular disease |
|  | Neighbors of stroke |
|  | Neighbors of cancer |
|  | Neighbors of cerebral ischemia |
|  | Neighbors of type 2 diabetes |
|  | Neighbors of neoplasm |
|  | Neighbors of endometriosis |
|  | Neighbors of neointima |
|  | Neighbors of vasculitis |
|  | Neighbors of atrophy |
|  | Neighbors of diet-induced obesity |
|  | Neighbors of respiratory hypersensitivity |
|  | Neighbors of myocardial fibrosis |
|  | Neighbors of synovitis |
|  | Neighbors of coronary artery disease |
|  | Neighbors of rupture |
|  | Neighbors of endothelial cell dysfunction |
|  | Neighbors of liver disease |
|  | Neighbors of prostate cancer |
|  | Neighbors of hypertrophy |
|  | Neighbors of osteolysis |
|  | Neighbors of ovarian cancer |
|  | Neighbors of pancreatic cancer |
|  | Neighbors of hypertension |
|  | Neighbors of intimal hyperplasia |
|  | Neighbors of allograft rejection |
|  | Neighbors of proteinuria |
|  | Neighbors of epithelial cell |
|  | Neighbors of keratinocyte |
|  | Neighbors of vascular smooth muscle cell |
|  | Neighbors of umbilical vein endothelial cell |
|  | Neighbors of monocyte |
|  | Neighbors of endometrial stromal cell |
|  | Neighbors of alveolar macrophage |
|  | Neighbors of synoviocyte |
|  | Neighbors of macrophage |
|  | Neighbors of adipocyte |
|  | Neighbors of activated monocyte |
|  | Neighbors of fibroblast |
|  | Neighbors of airway epithelial cell |
|  | Neighbors of PBMC |
|  | Neighbors of stromal cell |
|  | Neighbors of airway smooth muscle cell |
|  | Neighbors of osteoblast |
|  | Neighbors of pneumocyte |
|  | Neighbors of endometrium cell |
|  | Neighbors of microglia |
|  | Neighbors of trophoblast |
|  | Neighbors of intestine epithelium cell |
|  | Neighbors of mononuclear cell |
|  | Neighbors of hepatic stellate cell |
|  | Neighbors of hepatocyte |
|  | Neighbors of decidual cell |
|  | Neighbors of kidney mesangial cell |
|  | Neighbors of endothelial cell |
|  | Neighbors of dendritic cell |
|  | Neighbors of Kuppfer cell |
|  | Neighbors of proadipocyte |
|  | Neighbors of kidney tubule cell |
|  | Neighbors of inflammatory cell |
|  | Neighbors of bone marrow macrophage |
|  | Neighbors of peritoneal macrophage |
|  | Neighbors of osteoclast |
|  | Neighbors of neutrophil |
|  | Neighbors of smooth muscle myocyte |
|  | Neighbors of astrocyte |
|  | Neighbors of bone marrow derived dendritic cell |
|  | Neighbors of lung fibroblast |
|  | Neighbors of activated T-cell |
|  | Neighbors of mesothelial cell |
|  | Neighbors of mononuclear phagocyte |
|  | Neighbors of capillary endothelial cell |
|  | Neighbors of immunocompetent cell |
|  | Neighbors of granulocyte |
|  | Neighbors of cardiac myocyte |
|  | Neighbors of cancer-associated fibroblast |
|  | Neighbors of myeloid cell |
|  | Neighbors of mesenchymal stroma cell |
|  | Neighbors of corneal epithelial cell |
|  | Neighbors of stem cell |
|  | Neighbors of tumor infiltrating macrophage |
|  | Neighbors of sinusoidal cell |
|  | Neighbors of B-cell |
|  | Neighbors of granulosa cell |
|  | Neighbors of leukocyte |
|  | Neighbors of chondrocyte |
|  | Neighbors of insulin-secreting cell |
|  | Neighbors of mesenchymal cell |
|  | Neighbors of T-cell |
|  | Neighbors of M1 macrophage |
|  | Neighbors of myofibroblast |
|  | Neighbors of pancreatic stellate cell |
|  | Neighbors of mast cell |
|  | Neighbors of bone marrow stromal cell |
|  | Neighbors of spleen cell |
|  | Neighbors of brain capillary endothelial cell |
|  | Neighbors of neuroglia |
|  | Neighbors of thyroid epithelial cell |
|  | Neighbors of aortic smooth muscle cell |
|  | Neighbors of hematopoietic cell |
|  | Neighbors of adipose macrophage |
|  | Neighbors of podocyte |
|  | Neighbors of mammary epithelial cell |
|  | Neighbors of conventional dendritic cell |
|  | Neighbors of pericyte |
|  | Neighbors of antigen-presenting cell |
|  | Neighbors of epidermis cell |
|  | Neighbors of bone marrow derived mast cell |
|  | Neighbors of phagocyte |
|  | Neighbors of kidney cell |
|  | Neighbors of lymphocyte |
|  | Neighbors of Langerhans cell |
|  | Neighbors of decidual natural killer cell |
|  | Neighbors of thymic epithelial cell |
|  | Neighbors of brain cell |
|  | Neighbors of M2 macrophage |
|  | Neighbors of peritoneum cell |
|  | Neighbors of retina cell |
|  | Neighbors of blood cell |
|  | Neighbors of skin cell |
|  | Neighbors of luteal cell |
|  | Neighbors of peripheral lymphocyte |
|  | Neighbors of lymph node cell |
|  | Neighbors of lung |
|  | Neighbors of liver |
|  | Neighbors of kidney |
|  | Neighbors of skin |
|  | Neighbors of lymph node |
|  | Neighbors of colon |
|  | Neighbors of blood vessel |
|  | Neighbors of heart |
|  | Neighbors of aorta |
|  | Neighbors of gastrointestinal tract |
|  | Neighbors of microvessel |
|  | Neighbors of bone |
|  | Neighbors of placenta |
|  | Neighbors of synovial membrane |
|  | Neighbors of uterus |
|  | Neighbors of retina |
|  | Neighbors of brain |
|  | Neighbors of ovary |
|  | Neighbors of breast |
|  | Neighbors of blood vessel wall |
|  | Neighbors of islets of Langerhans |
|  | Neighbors of joint |
|  | Neighbors of artery |
|  | Neighbors of cardiovascular system |
|  | Neighbors of pancreas |
|  | Neighbors of prostate |
|  | Neighbors of central nervous system |
|  | Neighbors of kidney glomerulus |
|  | Neighbors of colorectal region |
|  | Neighbors of decidua |
|  | Neighbors of amnion |
|  | Neighbors of immune system |
|  | Neighbors of spleen |
|  | Neighbors of peritoneum |
|  | Neighbors of alveolus |
|  | Neighbors of coronary artery |
|  | Neighbors of uterine cervix |
|  | Neighbors of omentum |
|  | Neighbors of vein |
|  | Neighbors of arteriole |
|  | Neighbors of bronchus |
|  | Neighbors of nose |
|  | Neighbors of cornea |
|  | Neighbors of hippocampus |
|  | Neighbors of intervertebral disc |
|  | Neighbors of lymph node of mesentery |
|  | Neighbors of vagina |
|  | Neighbors of vitreous body |
|  | Neighbors of cerebral cortex |
|  | Neighbors of brain blood vessel |
|  | Neighbors of bladder |
|  | Neighbors of nervous system |
|  | Neighbors of retina blood vessel |
|  | Neighbors of cervical lymph node group |
|  | Neighbors of periodontal ligament |
|  | Neighbors of corpus luteum of ovary |
|  | Neighbors of nerve |
|  | Neighbors of globus pallidus |
|  | Neighbors of amniotic cavity |
|  | Neighbors of knee joint |
|  | Neighbors of lymphatic vessels |
|  | Neighbors of reproductive system |
|  | Neighbors of pleura |
|  | Neighbors of choroid plexus |
|  | Neighbors of hair follicle |
|  | Neighbors of artery wall |
|  | Neighbors of NF-kB family |
|  | Neighbors of oxidized LDL |
|  | Neighbors of JNK |
|  | Neighbors of IL1 family |
|  | Neighbors of PPAR |
|  | Neighbors of Jun/Fos |
|  | Neighbors of NFKBI |
|  | Neighbors of PKC |
|  | Neighbors of PI3K |
|  | Neighbors of STAT family |
|  | Neighbors of mitogen-activated protein kinase |
|  | Neighbors of IFNAR ligand |
|  | Neighbors of transforming growth factor |
|  | Neighbors of TLR |
|  | Neighbors of cytokine |
|  | Neighbors of interferon |
|  | Neighbors of JAK |
|  | Neighbors of MEK1/2 |
|  | Neighbors of PDGF |
|  | Neighbors of Ras GTPase |
|  | Neighbors of ERK1/2 |
|  | Neighbors of growth factor |
|  | Neighbors of PKA |
|  | Neighbors of ovalbumin |
|  | Neighbors of LDL |
|  | Neighbors of NF-AT family |
|  | Neighbors of beta adrenoceptor |
|  | Neighbors of chorionic gonadotropin |
|  | Neighbors of IKBK |
|  | Neighbors of adipokine |
|  | Neighbors of protein tyrosine kinase |
|  | Neighbors of NAD(P)H oxidase |
|  | Neighbors of IgG |
|  | Neighbors of SMAD subfamily |
|  | Neighbors of AMPK |
|  | Neighbors of LXR |
|  | Neighbors of HDL |
|  | Neighbors of p38 |
|  | Neighbors of inflammatory cytokine |
|  | Neighbors of IL23 |
|  | Neighbors of estrogen receptor |
|  | Neighbors of T-cell receptor |
|  | Neighbors of histone deacetylase |
|  | Neighbors of PDGF-BB |
|  | Neighbors of IL1R |
|  | Neighbors of HIF-1 |
|  | Neighbors of IL12 |
|  | Neighbors of Notch |
|  | Neighbors of prostaglandin-endoperoxide synthase |
|  | Neighbors of heat shock protein 90 |
|  | Neighbors of fibrinogen |
|  | Neighbors of aldehyde reductase |
|  | Neighbors of mitogen-activated protein kinase kinase |
|  | Neighbors of interleukin-35 |
|  | Neighbors of TSH |
|  | Neighbors of Ang II receptor |
|  | Neighbors of endothelin |
|  | Neighbors of NOS |
|  | Neighbors of superoxide dismutase |
|  | Neighbors of proteasome endopeptidase complex |
|  | Neighbors of class nuclear receptor with C4 zinc fingers |
|  | Neighbors of non-coding RNA |
|  | Neighbors of histone H3 |
|  | Neighbors of STAT5 |
|  | Neighbors of alpha2 adrenoceptor |
|  | Neighbors of retinoid-X receptor subfamily |
|  | Neighbors of SAPK |
|  | Neighbors of gonadotropins |
|  | Neighbors of immunoglobulin |
|  | Neighbors of PP2B |
|  | Neighbors of phospholipase |
|  | Neighbors of AHR subfamily |
|  | Neighbors of ITG |
|  | Neighbors of endostatins |
|  | Neighbors of relaxin |
|  | Neighbors of TORC1 |
|  | Neighbors of angiotensin receptor |
|  | Neighbors of RA receptor |
|  | Neighbors of histone |
|  | Neighbors of Rho kinase |
|  | Neighbors of PDGF receptor |
|  | Neighbors of neuropeptides |
|  | Neighbors of pancreatic elastase |
|  | Neighbors of adrenoceptor |
|  | Neighbors of phosphodiesterase |
|  | Neighbors of caspase |
|  | Neighbors of phosphoinositide phospholipase C |
|  | Neighbors of deacetylase |
|  | Neighbors of TLR4/Myd88 |
|  | Neighbors of TNF-alpha receptor |
|  | comon ps\_pos, pprarg\_neg targets |
|  | IFN signaling in alveolar cells |
|  | Overview. Diseases of circulatory system (chapter 8) |
|  | Overview. Diseases of the nervous system (chapter 5) |
|  | Overview. Diseases of the genitourinary system (chapter 13) |
|  | 2\_Interleukin-17 and interleukin-22 signaling in psoriasis |
|  | 1\_Low-density lipoprotein internalization and the accumulation of lipids in atherosclerosis |
|  | 2\_Oxidized LDL causes endothelial dysfunction in atherosclerosis |
|  | 3\_Role of alveolar macrophages in pulmonary tissue destruction in COPD |
|  | 2\_2\_Eosinophilia and neutrophilia in asthma: eosinophil apoptosis |
|  | 4\_1\_Polymorphisms associated with inflammatory bowel diseases |
|  | 4\_2\_Insulin resistance: inflammation-related insulin resistance |
|  | 4\_3\_Insulin resistance: FFA-related insulin resistance |
|  | 1\_Antigen-presenting cells promote islet dysfunction and immune system activation in T1D |
|  | 1\_Microangiopathy in diabetic neuropathy |
|  | drug-target pairs direct regulation |
|  | drug-target moltransport |
|  | Interleukin-17 and interleukin-22 signaling in psoriasis |

---

|  |  |
| --- | --- |
| Group | Genes with Mutations Associated with Atherosclerosis |
|  | Genes Associated with Systemic Lupus Erythematosus |
|  | Genes with Mutations Associated with Crohn's Disease |
|  | Genes with Mutations Associated with Systemic Scleroderma |

---

|  |  |
| --- | --- |
| Source | curated |
|  | GO component |

---

|  |  |
| --- | --- |
| MedScan ID | 6347 |
|  | 658850 |

---

|  |  |
| --- | --- |
| LocusLink ID | 6347 |
|  | 20293 |
|  | 287562 |
|  | 24770 |
|  | 103680 |
|  | 20296 |
|  | 793193 |
|  | 281043 |
|  | 282062 |

---

|  |  |
| --- | --- |
| Alias | C-C motif chemokine 12-like |
|  | HSMCR30 |
|  | Ccl12 |
|  | Monocyte chemoattractant protein-1 |
|  | chemokine (C-C motif) ligand 12 |
|  | Small inducible cytokine A2 |
|  | SY02\_HUMAN |
|  | small inducible cytokine A2 (monocyte chemotactic protein 1, homologous to mouse Sig-je) |
|  | MGC9434 |
|  | Monocyte chemotactic protein I |
|  | small inducible cytokine A2 (monocyte chemotactic protein 1) |
|  | monocyte chemotactic and activating factor |
|  | chemokine (C-C motif) ligand 2 |
|  | C-C motif chemokine ligand 2 |
|  | monocyte chemo-attractant protein-1 |
|  | chemokine (C-C motif) ligand II |
|  | GDCF II |
|  | Monocyte chemoattractant protein MCP-1 |
|  | small inducible cytokine A12 |
|  | LOC100504977 |
|  | Ccl-2 |
|  | Monocyte secretory protein JE |
|  | MCP-I |
|  | MCP-1/MCAF |
|  | MCP-1-related chemokine |
|  | CCL2 |
|  | SMC-CF |
|  | monocyte chemotactic protein 5 |
|  | small inducible cytokine subfamily A (Cys-Cys), member II |
|  | MCAF/MCP-1 |
|  | monocyte chemoattractant protein 5 |
|  | OTTMUSP00000000792 |
|  | MCP-1 |
|  | GDCF-2 |
|  | monocyte chemotactic protein 1, homologous to Sig-je |
|  | small inducible cytokine subfamily A (Cys-Cys), member 2 |
|  | SY02 |
|  | SCYA2 |
|  | MCP-5 |
|  | Monocyte chemotactic protein 1 |
|  | C-C motif chemokine 2 |
|  | C-C motif chemokine 12 |
|  | GDCF-2 HC11 |
|  | Scya12 |
|  | monocyte chemotactic protein 1, homologous to mouse Sig-je |
|  | GDCF IIs |
|  | LOC100048556 |
|  | Monocyte chemoattractant protein I |
|  | HC11 |
|  | MCAF |
|  | MCP1 |
|  | small-inducible cytokine A2 |
|  | small-inducible cytokine A12 |
|  | small inducible cytokine A2 (monocyte chemotactic protein 1, homologous to |
|  | AI323594 |
|  | monocyte chemoattractant protein 1 |
|  | Small inducible cytokine A2 (monocyte chemotactic protein, homologous to mo |
|  | monocyte chemotactic protein |
|  | Sigje |
|  | Ccl12\_predicted |
|  | Monocyte chemotactic and activating factor |
|  | HC 11 |
|  | monocyte chemotactic protein 1, homologous to Sig-je |
|  | HC11 protein |
|  | HC(11) |
|  | HC-11 |
|  | expressed sequence AI323594 |
|  | chemokine (C-C motif) ligand 12 (predicted) |
|  | LOC793193 |
|  | chromosome |
|  | hypothetical protein LOC793193 |
|  | scaffold |
|  | rp23-350g1.3 |
|  | small inducible gene je |
|  | homologous to mouse sig-je |

---

|  |  |
| --- | --- |
| GO ID | 0048020 |
|  | 0031727 |
|  | 0008009 |
|  | 0004672 |
|  | 0005102 |
|  | 0007186 |
|  | 0007187 |
|  | 0007259 |
|  | 0000165 |
|  | 0036499 |
|  | 0001525 |
|  | 0009887 |
|  | 0043615 |
|  | 0007155 |
|  | 0007166 |
|  | 0019725 |
|  | 0044344 |
|  | 0071346 |
|  | 0071347 |
|  | 0071222 |
|  | 0071407 |
|  | 0071356 |
|  | 0070098 |
|  | 0006935 |
|  | 0019221 |
|  | 0007010 |
|  | 0048245 |
|  | 0035684 |
|  | 0006959 |
|  | 0006954 |
|  | 0031663 |
|  | 0048247 |
|  | 0048246 |
|  | 0002548 |
|  | 2000134 |
|  | 0034351 |
|  | 2000502 |
|  | 0043524 |
|  | 1905563 |
|  | 0030593 |
|  | 0070374 |
|  | 0043547 |
|  | 1904783 |
|  | 0050870 |
|  | 2000427 |
|  | 0090280 |
|  | 2000353 |
|  | 0051770 |
|  | 0051968 |
|  | 0043491 |
|  | 0006468 |
|  | 0008360 |
|  | 0009617 |
|  | 0019233 |
|  | 0007165 |
|  | 0019079 |
|  | 0005623 |
|  | 0005576 |
|  | 0005615 |
|  | 0005125 |
|  | 0006955 |
|  | 0070664 |
|  | 0002687 |
|  | 0016004 |
|  | 0061844 |
|  | 0006816 |
|  | 0007267 |
|  | 0006874 |
|  | 0006887 |
|  | 0031640 |
|  | 0044828 |
|  | 0010469 |
|  | 0005622 |
|  | 0048471 |
|  | 0032967 |
|  | 0030425 |
|  | 0036006 |
|  | 0036120 |
|  | 0042493 |
|  | 0043066 |
|  | 0001666 |
|  | 0030139 |
|  | 0031100 |
|  | 0071403 |
|  | 0016525 |
|  | 0002523 |
|  | 0010759 |
|  | 0002693 |
|  | 0009408 |
|  | 0048010 |
|  | 0043085 |
|  | 0051384 |
|  | 0071549 |
|  | 0090314 |
|  | 0050729 |
|  | 0044299 |
|  | 0005737 |
|  | 0071300 |
|  | 0001938 |
|  | 0060137 |
|  | 0008201 |
|  | 0060135 |
|  | 0007568 |
|  | 0006916 |
|  | 0032760 |
|  | 0010574 |
|  | 0010332 |
|  | 0045202 |
|  | 0043025 |
|  | 0014823 |
|  | 0090026 |
|  | 0071318 |
|  | 0043679 |
|  | 0001912 |
|  | 0090265 |
|  | 0005791 |
|  | 0007179 |
|  | 0032869 |
|  | 0030968 |
|  | 0033552 |
|  | 0071354 |
|  | 0035690 |
|  | 0050806 |
|  | 0043200 |
|  | 0046677 |
|  | 0043204 |
|  | 0006987 |
|  | 0045471 |
|  | 0032570 |
|  | 0044267 |
|  | 0009612 |
|  | 0009611 |
|  | 0004871 |
|  | 0043277 |
|  | 0001664 |
|  | 0032496 |
|  | 0016020 |
|  | 0009618 |
|  | 0005515 |
|  | 0007600 |
|  | 0042466 |

---

|  |  |
| --- | --- |
| KEGG ID | hsa:6347 |
|  | mmu:20293 |
|  | mmu:105242482 |
|  | rno:287562 |

---

|  |  |
| --- | --- |
| MedLine Reference | 10331005 |
|  | 11751160 |
|  | 12029068 |
|  | 12071442 |
|  | 12080327 |
|  | 12133987 |
|  | 12135683 |
|  | 12196270 |
|  | 12370537 |
|  | 12372783 |
|  | 12377739 |
|  | 12390882 |
|  | 12409843 |
|  | 12540575 |
|  | 12540607 |
|  | 12761250 |
|  | 12870648 |
|  | 12899200 |
|  | 12950323 |
|  | 14505427 |
|  | 14638441 |
|  | 14684844 |
|  | 14693680 |
|  | 14967845 |
|  | 14978197 |
|  | 15178559 |
|  | 15183841 |
|  | 15205118 |
|  | 15240134 |
|  | 15308783 |
|  | 15504940 |
|  | 15627719 |
|  | 15742444 |
|  | 15829559 |
|  | 15880558 |
|  | 15917991 |
|  | 15930146 |
|  | 15978006 |
|  | 16102724 |
|  | 16116069 |
|  | 16127145 |
|  | 16132691 |
|  | 16164699 |
|  | 16174730 |
|  | 16196033 |
|  | 16265704 |
|  | 16282363 |
|  | 16697654 |
|  | 16799229 |
|  | 16886721 |
|  | 1704226 |
|  | 10079097 |
|  | 10349636 |
|  | 10395330 |
|  | 10397169 |
|  | 10438970 |
|  | 10570305 |
|  | 10746730 |
|  | 10790402 |
|  | 10995569 |
|  | 11042159 |
|  | 11076861 |
|  | 11130978 |
|  | 11217851 |
|  | 11257138 |
|  | 11485904 |
|  | 11714821 |
|  | 11716961 |
|  | 11735217 |
|  | 11756151 |
|  | 11756344 |
|  | 11918087 |
|  | 11937571 |
|  | 11971015 |
|  | 12011011 |
|  | 12080938 |
|  | 12085314 |
|  | 12098066 |
|  | 12106688 |
|  | 12126650 |
|  | 12131093 |
|  | 12151080 |
|  | 12165826 |
|  | 12355436 |
|  | 12388376 |
|  | 12466851 |
|  | 12477932 |
|  | 12525571 |
|  | 12594253 |
|  | 12623845 |
|  | 12682245 |
|  | 12707348 |
|  | 12734374 |
|  | 12756299 |
|  | 12847259 |
|  | 12850812 |
|  | 12925209 |
|  | 13129912 |
|  | 1347470 |
|  | 1406651 |
|  | 1448065 |
|  | 14500652 |
|  | 14516793 |
|  | 14520519 |
|  | 14527928 |
|  | 14532907 |
|  | 14550294 |
|  | 14576355 |
|  | 14615370 |
|  | 14633605 |
|  | 14635040 |
|  | 14662884 |
|  | 14670305 |
|  | 14679181 |
|  | 14688348 |
|  | 14709405 |
|  | 14739122 |
|  | 15019295 |
|  | 15020650 |
|  | 15033992 |
|  | 15063799 |
|  | 15075201 |
|  | 15081318 |
|  | 15095371 |
|  | 15153515 |
|  | 15163738 |
|  | 15210789 |
|  | 15240672 |
|  | 15358037 |
|  | 15377395 |
|  | 15381731 |
|  | 15518805 |
|  | 15528472 |
|  | 15539456 |
|  | 15579457 |
|  | 15579460 |
|  | 15597153 |
|  | 15611273 |
|  | 15661915 |
|  | 15705791 |
|  | 15712606 |
|  | 15718500 |
|  | 15748167 |
|  | 15774854 |
|  | 15829914 |
|  | 15855647 |
|  | 15886005 |
|  | 15928200 |
|  | 16014038 |
|  | 16105698 |
|  | 16115031 |
|  | 16129712 |
|  | 16141072 |
|  | 16141073 |
|  | 16166627 |
|  | 16166738 |
|  | 16177325 |
|  | 16239969 |
|  | 16243300 |
|  | 16278381 |
|  | 16299331 |
|  | 16337133 |
|  | 16339568 |
|  | 16339576 |
|  | 16397233 |
|  | 16443645 |
|  | 16462739 |
|  | 16678847 |
|  | 16691201 |
|  | 16691291 |
|  | 16728728 |
|  | 16809344 |
|  | 16825596 |
|  | 16882009 |
|  | 16888027 |
|  | 16960104 |
|  | 16982835 |
|  | 16990567 |
|  | 16997857 |
|  | 17047515 |
|  | 17074847 |
|  | 17112893 |
|  | 17142777 |
|  | 1794049 |
|  | 1969921 |
|  | 2004761 |
|  | 2065676 |
|  | 2071154 |
|  | 2513477 |
|  | 2576529 |
|  | 2910858 |
|  | 3108379 |
|  | 3287374 |
|  | 6872001 |
|  | 7528168 |
|  | 7579889 |
|  | 7590247 |
|  | 7594550 |
|  | 7601457 |
|  | 7622488 |
|  | 7642742 |
|  | 7650394 |
|  | 7717454 |
|  | 7737353 |
|  | 7767000 |
|  | 7789623 |
|  | 7836768 |
|  | 7945333 |
|  | 7947467 |
|  | 7954525 |
|  | 8051420 |
|  | 8132324 |
|  | 8144933 |
|  | 8216854 |
|  | 8268655 |
|  | 8342023 |
|  | 8423056 |
|  | 8449498 |
|  | 8455595 |
|  | 8631787 |
|  | 8661152 |
|  | 8662231 |
|  | 8683100 |
|  | 8757628 |
|  | 8759763 |
|  | 8827514 |
|  | 8835527 |
|  | 8920881 |
|  | 8981365 |
|  | 8996246 |
|  | 9098403 |
|  | 9177770 |
|  | 9192769 |
|  | 9196007 |
|  | 9321482 |
|  | 9365109 |
|  | 9366570 |
|  | 9405660 |
|  | 9463410 |
|  | 9480983 |
|  | 9653646 |
|  | 9734366 |
|  | 9779377 |
|  | 9786242 |
|  | 9990422 |
|  | 10068657 |
|  | 10198043 |
|  | 10453044 |
|  | 10477718 |
|  | 10529171 |
|  | 10542238 |
|  | 10623817 |
|  | 10644332 |
|  | 10706668 |
|  | 10734104 |
|  | 10751368 |
|  | 10770925 |
|  | 10942204 |
|  | 10982368 |
|  | 10985244 |
|  | 11083865 |
|  | 11145723 |
|  | 11154208 |
|  | 11750041 |
|  | 11751881 |
|  | 11776402 |
|  | 11912219 |
|  | 11989790 |
|  | 11999660 |
|  | 12009356 |
|  | 12022754 |
|  | 12067898 |
|  | 12078856 |
|  | 12082252 |
|  | 12093796 |
|  | 12117737 |
|  | 12127997 |
|  | 12149192 |
|  | 12183528 |
|  | 12186702 |
|  | 12195705 |
|  | 12204371 |
|  | 12234797 |
|  | 12239249 |
|  | 12351486 |
|  | 12372466 |
|  | 12374865 |
|  | 12391099 |
|  | 12391196 |
|  | 12393171 |
|  | 12397639 |
|  | 12399623 |
|  | 12408680 |
|  | 12410798 |
|  | 12413001 |
|  | 12419245 |
|  | 12443832 |
|  | 12460032 |
|  | 12470469 |
|  | 12473373 |
|  | 12485413 |
|  | 12488502 |
|  | 12505750 |
|  | 12520153 |
|  | 12520365 |
|  | 12540965 |
|  | 12632067 |
|  | 12665582 |
|  | 12724308 |
|  | 12753088 |
|  | 12758167 |
|  | 12787135 |
|  | 12805068 |
|  | 12826189 |
|  | 12826377 |
|  | 12846738 |
|  | 12946945 |
|  | 12957789 |
|  | 12960255 |
|  | 13679391 |
|  | 14517168 |
|  | 14517792 |
|  | 14550782 |
|  | 14576080 |
|  | 14597108 |
|  | 14602575 |
|  | 14620921 |
|  | 14660607 |
|  | 14687706 |
|  | 14733721 |
|  | 15016614 |
|  | 15022320 |
|  | 15034225 |
|  | 15041705 |
|  | 15077296 |
|  | 15082170 |
|  | 15110609 |
|  | 15123743 |
|  | 15167690 |
|  | 15188361 |
|  | 15191525 |
|  | 15191888 |
|  | 15191916 |
|  | 15203564 |
|  | 15226634 |
|  | 15257681 |
|  | 15280531 |
|  | 15312962 |
|  | 15349727 |
|  | 15389752 |
|  | 15466648 |
|  | 15492853 |
|  | 15529362 |
|  | 15545821 |
|  | 15599324 |
|  | 15602730 |
|  | 15611878 |
|  | 15654958 |
|  | 15677312 |
|  | 15722361 |
|  | 15745922 |
|  | 15780578 |
|  | 15832427 |
|  | 15866653 |
|  | 15880317 |
|  | 15883814 |
|  | 15900302 |
|  | 15919935 |
|  | 15976326 |
|  | 15979992 |
|  | 16003740 |
|  | 16004967 |
|  | 16009177 |
|  | 16009638 |
|  | 16020745 |
|  | 16095529 |
|  | 16105652 |
|  | 16129702 |
|  | 16195477 |
|  | 16204411 |
|  | 16206198 |
|  | 16213182 |
|  | 16214031 |
|  | 16230097 |
|  | 16249002 |
|  | 16280328 |
|  | 16280979 |
|  | 16307829 |
|  | 16309586 |
|  | 16318581 |
|  | 16351713 |
|  | 16352737 |
|  | 16358960 |
|  | 16359995 |
|  | 16362156 |
|  | 16415174 |
|  | 16427785 |
|  | 16436595 |
|  | 16439461 |
|  | 16445900 |
|  | 16474202 |
|  | 16479072 |
|  | 16504689 |
|  | 16516290 |
|  | 16518346 |
|  | 16597919 |
|  | 16609683 |
|  | 1661560 |
|  | 16631114 |
|  | 16636247 |
|  | 16636603 |
|  | 16672419 |
|  | 16697212 |
|  | 16705739 |
|  | 16712788 |
|  | 16730843 |
|  | 16733654 |
|  | 16751386 |
|  | 16781696 |
|  | 16795034 |
|  | 16802342 |
|  | 16825597 |
|  | 16828028 |
|  | 16867220 |
|  | 16873204 |
|  | 16939660 |
|  | 16980310 |
|  | 16997300 |
|  | 17052731 |
|  | 17082648 |
|  | 17098977 |
|  | 17187019 |
|  | 17202846 |
|  | 2357211 |
|  | 2465924 |
|  | 2501656 |
|  | 2518726 |
|  | 2923622 |
|  | 7651403 |
|  | 8051410 |
|  | 8107690 |
|  | 8195247 |
|  | 8639605 |
|  | 8989326 |
|  | 9287323 |
|  | 9364936 |
|  | 9405404 |
|  | 9501225 |
|  | 9548499 |
|  | 9558113 |
|  | 9670957 |
|  | 9789057 |
|  | 9792375 |
|  | 9973507 |
|  | 10587439 |
|  | 10918580 |
|  | 15489334 |
|  | 16814297 |
|  | 16835702 |
|  | 17008880 |
|  | 17032917 |
|  | 17065190 |
|  | 17091019 |
|  | 17138827 |
|  | 17169533 |
|  | 17218539 |
|  | 17262819 |
|  | 17280738 |
|  | 17283105 |
|  | 17284607 |
|  | 17307163 |
|  | 17322498 |
|  | 17364892 |
|  | 17379849 |
|  | 17384985 |
|  | 17390372 |
|  | 17395051 |
|  | 17404035 |
|  | 17407231 |
|  | 17424890 |
|  | 17458901 |
|  | 17465499 |
|  | 17485071 |
|  | 17495598 |
|  | 17522215 |
|  | 17525255 |
|  | 17530707 |
|  | 17596666 |
|  | 1857712 |
|  | 2322286 |
|  | 2648385 |
|  | 8627182 |
|  | 8898111 |
|  | 9792674 |
|  | 9837883 |
|  | 11980865 |
|  | 14662900 |
|  | 16543609 |
|  | 16873893 |
|  | 17003237 |
|  | 17088347 |
|  | 17125873 |
|  | 17126305 |
|  | 17135576 |
|  | 17160673 |
|  | 17220320 |
|  | 17258864 |
|  | 17283277 |
|  | 17307812 |
|  | 17329566 |
|  | 17347584 |
|  | 17364026 |
|  | 17371458 |
|  | 17409491 |
|  | 17433708 |
|  | 17452470 |
|  | 17456776 |
|  | 17473219 |
|  | 17507908 |
|  | 17549399 |
|  | 17575105 |
|  | 17589343 |
|  | 17640882 |
|  | 17652758 |
|  | 17662241 |
|  | 17710158 |
|  | 9884336 |
|  | 16758320 |
|  | 16968890 |
|  | 17164575 |
|  | 17180354 |
|  | 17191078 |
|  | 17303665 |
|  | 17460389 |
|  | 17530714 |
|  | 17581219 |
|  | 17632282 |
|  | 2106664 |
|  | 1543494 |
|  | 15659710 |
|  | 1721821 |
|  | 8060303 |

---

|  |  |
| --- | --- |
| Organism | Homo sapiens {Organism urn:agi-taxid:9606} |
|  | Mus musculus {Organism urn:agi-taxid:10090} |
|  | Rattus norvegicus {Organism urn:agi-taxid:10116} |
|  | Homo sapiens |
|  | Mus musculus |
|  | Rattus norvegicus |
|  | Danio rerio |
|  | Bos taurus |

---

|  |  |
| --- | --- |
| Mouse chromosome position | 11 49.9 cM |
|  | 11 47.0 cM |

---

|  |  |
| --- | --- |
| OMIM ID | 158105 |
|  | 609423 |
|  | 607948 |
|  | 182940 |

---

|  |  |
| --- | --- |
| Rat chromosome position | 10q26 |

---

|  |  |
| --- | --- |
| Hugo ID | 10618 |
|  | HGNC:10618 |

---

|  |  |
| --- | --- |
| Human chromosome position | 17q12 |
|  | 17q11.2-q12 |

---

|  |  |
| --- | --- |
| Swiss-Prot Accession | P13500 |
|  | P13500.1 |
|  | Q545B5 |
|  | Q62401 |
|  | Q62401.1 |
|  | D4ABS1 |
|  | B2R4V3 |
|  | Q9UDF3 |
|  | Q9QYD6 |
|  | Q6UZ82 |
|  | Q5SVB4 |
|  | Q545B5\_MOUSE |
|  | CCL12\_MOUSE |
|  | CCL2\_HUMAN |
|  | B2R4V3\_HUMAN |

---

|  |  |
| --- | --- |
| PIR ID | A35474 |

---

|  |  |
| --- | --- |
| GenBank ID | NC\_000017 |
|  | NM\_002982 |
|  | NP\_002973 |
|  | NG\_012123 |
|  | AC005549 |
|  | AF519531 |
|  | AAM54046 |
|  | AH001509 |
|  | AAA36330 |
|  | AH002970 |
|  | AAA60308 |
|  | AY357296 |
|  | AAQ75526 |
|  | CH471147 |
|  | EAW80211 |
|  | EAW80212 |
|  | D26087 |
|  | BAA05080 |
|  | M37719 |
|  | AAA18102 |
|  | X60001 |
|  | Y18933 |
|  | CAC14049 |
|  | AK311960 |
|  | BAG34900 |
|  | AV733621 |
|  | BC009716 |
|  | AAH09716 |
|  | BG530064 |
|  | BT007329 |
|  | AAP35993 |
|  | M24545 |
|  | AAA18164 |
|  | M26683 |
|  | AAA96708 |
|  | M28226 |
|  | AAA60309 |
|  | S69738 |
|  | AAB29926 |
|  | S71513 |
|  | AAB20651 |
|  | X14768 |
|  | CAA32876 |
|  | P13500 |
|  | NC\_000077 |
|  | NM\_011331 |
|  | NP\_035461 |
|  | AL645596 |
|  | DS033261 |
|  | EDL01207 |
|  | DS033265 |
|  | EDL01219 |
|  | AF065934 |
|  | AAF15384 |
|  | AF065935 |
|  | AAF15385 |
|  | AF065936 |
|  | AAF15386 |
|  | AF065937 |
|  | AAF15387 |
|  | AF065938 |
|  | AAF15388 |
|  | AK012356 |
|  | BAB28185 |
|  | BC027520 |
|  | AAH27520 |
|  | U50712 |
|  | AAB50053 |
|  | U66670 |
|  | AAB49424 |
|  | Q62401 |
|  | NC\_005109 |
|  | NM\_001105822 |
|  | NP\_001099292 |
|  | AC\_000078 |
|  | AAHX01065149 |
|  | AC114440 |
|  | CH473948 |
|  | EDM05440 |
|  | ABM84651 |
|  | ABW03626 |
|  | EU176175 |
|  | AAHY01094593 |
|  | AAHY01094594 |
|  | AC\_000149 |
|  | CAI25041 |
|  | AC\_000033 |
|  | DQ893725 |
|  | AMYH02033336 |
|  | AABR06064880 |
|  | ABBA01011212 |
|  | NC\_018928 |
|  | M31625 |
|  | M31626 |
|  | M30816 |
|  | M28225 |
|  | M28224 |
|  | M28223 |
|  | NT\_010799 |
|  | NW\_001838432 |
|  | NW\_926772 |
|  | AC\_000060 |
|  | Q6UZ82 |
|  | NT\_096135 |
|  | NW\_001030484 |
|  | NW\_001084656 |
|  | NW\_047336 |
|  | NW\_001030485 |
|  | XM\_001480891 |
|  | XP\_001480941 |
|  | XM\_001480892 |
|  | XP\_001480942 |
|  | XM\_001480893 |
|  | XP\_001480943 |
|  | XM\_001081016 |
|  | XP\_213425 |
|  | XM\_213425 |
|  | XP\_001081016 |
|  | Q5SVB4 |
|  | BAE29972 |
|  | J04467 |
|  | CAA34901 |
|  | BAE30690 |
|  | AF065929 |
|  | BAE21250 |
|  | BAE31998 |
|  | M19681 |
|  | CAJ18395 |
|  | AAA37684 |
|  | NP\_035463 |
|  | NW\_001030424 |
|  | NM\_011333 |
|  | BC055070 |
|  | CAI25305 |
|  | BAE32062 |
|  | NW\_000039 |
|  | P10148 |
|  | CT010187 |
|  | BAE32019 |
|  | 126846 |
|  | 205334 |
|  | 3091141 |
|  | 55531 |
|  | 81873033 |
|  | AAA63496 |
|  | AAC15187 |
|  | AF058786 |
|  | AF079313 |
|  | M57441 |
|  | NM\_031530 |
|  | NP\_113718 |
|  | P14844 |
|  | Q549R5 |
|  | X17053 |
|  | 123790955 |
|  | 123797015 |
|  | 126844 |
|  | 32822882 |
|  | 387168 |
|  | 387169 |
|  | 56205230 |
|  | 6531363 |
|  | 6531365 |
|  | 6531367 |
|  | 6531369 |
|  | 6531371 |
|  | 71059703 |
|  | 74142494 |
|  | 74151961 |
|  | 74152056 |
|  | 74181396 |
|  | 74198942 |
|  | 74201832 |
|  | 81870303 |
|  | 81885950 |
|  | AAA37685 |
|  | AAF15379 |
|  | AAF15380 |
|  | AAF15381 |
|  | AAF15382 |
|  | AAF15383 |
|  | AAH55070 |
|  | AF065930 |
|  | AF065931 |
|  | AF065932 |
|  | AF065933 |
|  | AJ238892 |
|  | AK132590 |
|  | AK150937 |
|  | AK151789 |
|  | AK153443 |
|  | AK153468 |
|  | AK153520 |
|  | AL626807 |
|  | Q3U5L6 |
|  | Q3U5S8 |
|  | Q5SVU3 |
|  | Q7TMS1 |
|  | AI323594 |
|  | 10933861 |
|  | 119600618 |
|  | 123994099 |
|  | 126842 |
|  | 16307255 |
|  | 184642 |
|  | 21435977 |
|  | 240868 |
|  | 30583497 |
|  | 307163 |
|  | 338007 |
|  | 338009 |
|  | 34514 |
|  | 34559720 |
|  | 386961 |
|  | 487124 |
|  | 545465 |
|  | 74738319 |
|  | 992947 |
|  | NT\_086870 |
|  | NT\_110624 |
|  | 240867 |
|  | 51828859 |
|  | 6755429 |
|  | 6755430 |
|  | 17902508 |
|  | 193488 |
|  | 193486 |
|  | 6531362 |
|  | 6531364 |
|  | 6531366 |
|  | 6531368 |
|  | 6531370 |
|  | 34873416 |
|  | 13928713 |
|  | 13928714 |
|  | 3388147 |
|  | 55530 |
|  | 3091140 |
|  | 205333 |
|  | 34513 |
|  | 37544509 |
|  | 56119169 |
|  | 4506841 |
|  | 51474487 |
|  | 21435976 |
|  | 34559719 |
|  | 516772 |
|  | 338005 |
|  | 188701 |
|  | 187447 |
|  | 34001 |
|  | 10933860 |
|  | 55950752 |
|  | 16307254 |
|  | 30583496 |
|  | 187434 |
|  | 184641 |
|  | 338008 |
|  | 545464 |
|  | NC\_007131 |
|  | NW\_001512140 |
|  | XM\_001332867 |
|  | XP\_001332903 |
|  | 119600617 |
|  | 148683709 |
|  | 148683710 |
|  | 148877642 |
|  | 148877899 |
|  | AAI45868 |
|  | AAI45870 |
|  | BC145867 |
|  | BC145869 |
|  | CH466556 |
|  | EDL15656 |
|  | EDL15657 |
|  | 149053620 |
|  | EDM05437 |
|  | 126841 |
|  | 148743988 |
|  | 163395 |
|  | 624394 |
|  | AAA30651 |
|  | AAA60956 |
|  | AAI42094 |
|  | BC142093 |
|  | L32659 |
|  | M84602 |
|  | NC\_007317 |
|  | NM\_174006 |
|  | NP\_776431 |
|  | NW\_001493657 |
|  | P28291 |
|  | 125846719 |
|  | 126007383 |
|  | 793193 |
|  | Dr.139901 |
|  | dre:793193 |

---

|  |  |
| --- | --- |
| Swiss-Prot ID | CCL2\_HUMAN |
|  | CCL12\_MOUSE |
|  | D4ABS1\_RAT |
|  | Q545B5\_MOUSE |
|  | B2R4V3\_HUMAN |

---

|  |  |
| --- | --- |
| Cell Localization | Secreted |
|  | Extracellular |

---

|  |  |
| --- | --- |
| Ensembl ID | ENSG00000108691 |
|  | ENSP00000225831.4 |
|  | ENST00000225831.4 |
|  | ENSMUSG00000035352 |
|  | ENSMUSP00000000194.3 |
|  | ENSMUST00000000194.3 |
|  | ENSRNOG00000029768 |
|  | ENSRNOP00000038407.4 |
|  | ENSRNOT00000031626.5 |
|  | ENST00000225831 |
|  | ENSP00000225831 |
|  | ENSMUST00000000194 |
|  | ENSRNOT00000031626 |
|  | ENSRNOP00000038407 |
|  | ENSMUSP00000000194 |

---

|  |  |
| --- | --- |
| MGI ID | MGI:108224 |
|  | 108224 |
|  | 98259 |
|  | 2144120 |

---

|  |  |
| --- | --- |
| RGD ID | 1309255 |
|  | 3645 |

---

|  |  |
| --- | --- |
| Unigene ID | Hs.303649 |
|  | Mm.867 |
|  | Rn.137780 |
|  | Rn.4772 |
|  | Mm.290320 |
|  | Dr.139901 |
|  | Bt.2408 |

---

|  |  |
| --- | --- |
| Homologene ID | 2245 |

---

|  |  |
| --- | --- |
| Shape | Rhomb |

---

|  |  |
| --- | --- |
| IPI ID | IPI00009308 |
|  | IPI00369956 |
|  | IPI00788881 |
|  | IPI00125860 |
|  | IPI00850699 |
|  | IPI00850528 |

---

|  |  |
| --- | --- |
| Chromosome position | 10q26 |
|  | 17q11.2-q21.1 |
|  | 11 46.5 cM |

---

|  |  |
| --- | --- |
| Microarray ID | 1367973\_at |
|  | x17053cds\_s\_at |
|  | A\_42\_P695401 |
|  | 102736\_at |
|  | 1420380\_at |
|  | m19681\_s\_at |
|  | A\_51\_P286737 |
|  | M26683 |
|  | M37719 |
|  | M24545 |
|  | 216598\_s\_at |
|  | 34375\_at |
|  | 875\_g\_at |
|  | M26683\_at |
|  | A\_23\_P89431 |
|  | A\_14\_P125482 |
|  | OR2485 |
|  | 202 |
|  | OH3487A |
|  | MA418 |
|  | AH1405 |
|  | SC169 |
|  | x17053mrna\_s\_at |
|  | 93717\_at |

---

|  |  |
| --- | --- |
| Homo sapiens Chromosome position | 17q11.2-q12 |

---

|  |  |
| --- | --- |
| Ensembl Transcript ID | ENST00000225831 |

---

|  |  |
| --- | --- |
| FunctionalClass | CCR2 chemokine receptor binding |
|  | G-protein-coupled receptor binding |
|  | chemokine |
|  | protein binding |
|  | protein kinase |
|  | cytokine |
|  | signal transducer |

---

|  |  |
| --- | --- |
| Hugo Symbol | CCL2 |

---

|  |  |
| --- | --- |
| Bos taurus Chromosome position | 19 |

---

|  |  |
| --- | --- |
| Mus musculus Chromosome position | 11 46.5 cM |
|  | 11 |

---

|  |  |
| --- | --- |
| KEGG pathway | Cytokine-cytokine receptor interaction |

---

|  |  |
| --- | --- |
| Rattus norvegicus Chromosome position | 10q26 |

---

|  |  |
| --- | --- |
| Ensembl Gene ID | ENSG00000108691 |

---
